# Electrical Retrieval of Living Microorganisms from Cryopreserved Marine Sponges Using a Potential-Controlled Electrode

**DOI:** 10.1007/s10126-015-9651-y

**Published:** 2015-08-05

**Authors:** Sumihiro Koyama, Shinro Nishi, Maki Tokuda, Moeka Uemura, Yoichi Ishikawa, Takeshi Seya, Seinen Chow, Yuji Ise, Yuji Hatada, Yoshihiro Fujiwara, Taishi Tsubouchi

**Affiliations:** Department of Marine Biodiversity Research, Japan Agency for Marine-Earth Science and Technology, 2-15 Natsushima-cho, Yokosuka, Kanagawa 237-0061 Japan; Research and Development Center for Marine Biosciences, Japan Agency for Marine-Earth Science and Technology, 2-15 Natsushima-cho, Yokosuka, Kanagawa 237-0061 Japan; Able Co. Ltd., 6-10 Nishigoken-cho, Shinjuku-ku, Tokyo 162-0812 Japan; Analog Technology Co. Ltd., 2-1-13 Motoyokoyama-cho, Hachioji, Tokyo 192-0063 Japan; National Research Institute of Fisheries Science, Fukuura 2-12-4, Kanazawa, Yokohama, Kanagawa 236-8648 Japan; Sugashima Marine Biological Laboratory, Nagoya University Graduate School of Science, 429-63 Sugashima, Toba, Mie 517-0004 Japan

**Keywords:** *Spirastrella insignis*, *Callyspongia confoederata*, Sponge, Symbiont, Electrical retrieval, Next-generation sequencing technology

## Abstract

The purpose of this study was to develop a novel electrical retrieval method (ER method) for living sponge-associated microorganisms from marine sponges frozen at −80 °C. A −0.3-V vs. Ag/AgCl constant potential applied for 2 h at 9 °C induced the attachment of the sponge-associated microorganisms to an indium tin oxide/glass (ITO) or a gallium-doped zinc oxide/glass (GZO) working electrode. The electrically attached microorganisms from homogenized *Spirastrella insignis* tissues had intact cell membranes and showed intracellular dehydrogenase activity. Dead microorganisms were not attracted to the electrode when the homogenized tissues were autoclaved for 15 min at 121 °C before use. The electrically attached microorganisms included cultivable microorganisms retrieved after detachment from the electrode by application of a 9-MHz sine-wave potential. Using the ER method, we obtained 32 phyla and 72 classes of bacteria and 3 archaea of *Crenarchaeota thermoprotei*, *Marine Group I*, and *Thaumarchaeota incertae sedis* from marine sponges *S. insignis* and *Callyspongia confoederata*. Employment of the ER method for extraction and purification of the living microorganisms holds potential of single-cell cultivation for genome, transcriptome, proteome, and metabolome analyses of bioactive compounds producing sponge-associated microorganisms.

## Introduction

Since marine sponges are a rich source of biologically active compounds with antitumor, antiviral, antibacterial, antifungal, antimalarial, antiprotozoal, and antituberculous activities, extensive effort has been made to isolate those compounds in the quest to find excellent drug candidates (Munro et al. 1999; Faulkner 2002; Imhoff and Stöhr 2003; Hentschel et al. 2003, 2012; Taylor et al. 2007; Mayer et al. 2013; Fuerst 2014). There is increasing evidence that some biologically active compounds appear to be produced by sponge-associated microorganisms (Bewley and Faulkner 1998; Haygood et al. 1999; Piel et al. 2004; Schmidt et al. 2000; Wilson et al. 2014). Marine sponges are sessile animals with no or little mechanical defense and rely heavily on the production of biologically active compounds as a form of defense against natural enemies, such as predators and competitors (Imhoff and Stöhr 2003; Taylor et al. 2007). It is apparently a widespread strategy among marine animals to acquire these compounds from symbiotic microorganisms as a chemical defense, such as tetrodotoxin production in the blue-ringed octopus and puffer fish (Hwang et al. 1989; Lee et al. 2000) and bryostatin production in bryozoans (Davidson et al. 2001; Lopanik et al. 2004). The production of chemical compounds by sponge-associated microorganisms as a defense strategy is important in the search for drug candidates because these chemicals are not often developed due to a variety of problems such as the rare occurrence of marine sponges, difficulties in recovery, and difficulties in reproduction (Imhoff and Stöhr 2003).

The cultivation of sponge-associated microorganisms that produce biologically active compounds is the most direct method for large-scale production of these chemicals (Hill 2004), and extensive cultivation approaches have been attempted by groups targeting bioactive compounds (Burja et al. 1999; Sponga et al. 1999; Höller et al. 2000; Jensen and Fenical 2000; Webster and Hill 2001; Burja and Hill 2001; Hentschel et al. 2001; Hill 2004; Selvin et al. 2004; Gunasekera et al. 2005; Montalvo et al. 2005; Dieckmann et al. 2005; Sfanos et al. 2005; Kim et al. 2006; Kennedy et al. 2009; Sipkema et al. 2011; Hosoya et al. 2012; Yamazaki et al. 2012; Graça et al. 2013; Izumikawa et al. 2013; Dashti et al. 2014; Pandey et al. 2014; Steinert et al. 2014). Although several researchers have reported improved cultivation of sponge-associated microorganisms using methods such as supplementing the media with sponge tissue extracts (Webster et al. 2001), catalase, and sodium pyruvate (Olson et al. 2000), only a minor fraction of the total sponge-associated microbial community was amenable to cultivation on laboratory media (Santavy et al. 1990; Burja et al. 1999; Webster and Hill 2001; Friedrich et al. 2001; Hill 2004). Many of the sponge associates are extremely difficult to obtain in pure culture due to nutritional or other dependencies (Taylor et al. 2007). Santavy et al. (1990) were able to achieve better recovery and estimated that 3 to 11 % of the total bacterial population from Caribbean sclerosponge *Ceratoporella nicholsoni* were cultured. Furthermore, some sponge-associated microorganisms simply stop producing the biologically active compounds after a certain time on artificial media (Hentschel et al. 2003). Therefore, cultivation of the sponge-associated microorganisms with varied compositions has been performed immediately after collection in order to screen for the diverse biologically active compounds (Burja et al. 1999; Burja and Hill 2001; Webster and Hill 2001; Montalvo et al. 2005; Sipkema et al. 2011). In streptomycete bacteria, many gene clusters that direct the biosynthesis of natural products with clinical potential are not expressed or at a very low level (Aigle and Corre 2012). Several researchers reported that genetically modified microorganisms improved the production of bioactive compounds by enhancement of precursor biosynthesis (Aigle and Corre 2012; Huang et al. 2013). Similarly, disrupting a negative pathway-specific regulator can also result in overproduction of the compounds (Aigle and Corre 2012; Huang et al. 2013). For example, improved production of an immunosuppressant FK506 has been succeeded in *gdhA*-deleted and *dahp*-, *accA2*-, and *zwf2*-overexpressed *Streptomyces tsukubaensis* (Huang et al. 2013). To find and improve the production of bioactive compounds from the sponge-associated microorganisms, it is important to analyze and compare a small amount of the intracellular metabolites before and after each of the cells stops producing the compounds in the culture. Therefore, it is necessary to obtain highly pure living sponge-associated microorganisms directly from homogenized marine sponges without the cultivation.

In our previous studies, we demonstrated that a weak negative electric potential attracted living microorganisms to the electrode surface (Koyama et al. 2013). Microorganisms such as *Escherichia coli* recognized small regions of the negative applied potential microelectrode and selectively attached to the 5-μmϕ circular microelectrode array (Koyama et al. 2013). We collected the electrically attached living microorganisms and then detached them from the electrode by applying a high-frequency wave potential (Koyama et al. 2013). The mechanisms for electrical detachment involve both oscillation of the negative zeta potential-charged microorganisms and insertion of water molecules between the electrode surface and the attached cells resulting from increments in hydrophilicity on the electrode surface (Koyama 2011; Koyama et al. 2013). When we applied the electrical retrieval (ER) method to separate the microorganisms from sediment and soil particles, bacteria belonging to 19 phyla and 23 classes were collected without undesirable high molecular weight contaminants such as humic acids (Koyama et al. 2013).

In the present study, we investigated whether the weak negative electric potential attracted sponge-associated living microorganisms to the electrode surface from marine sponges frozen at −80 °C. Moreover, we also examined whether the electrically retrieved living microorganisms are cultivable on an originally formulated agar medium.

## Materials and Methods

### Marine Sponge Collection

The marine sponges *Spirastrella insignis* and *Callyspongia confoederata* were collected by hand at a depth of 2 m in the ocean near the Miura Peninsula, Japan (35° 11′ N, 139 ° 36′ E; collection date: March 25, 2014). The fresh specimens were minced with a knife and transferred to 50-mL plastic tubes at the underwater collection sites. We made and analyzed specimens of marine sponge spicules under a microscope (Hooper and Van Soest 2002). Examinations of the general morphological features including the geometry of spicules of the each tissue sample classified the marine sponges *S. insignis* (Thiele 1898; Hooper and Van Soest 2002) and *C. confoederata* (Ridley 1884; Hooper and Van Soest 2002), respectively. Some of the collected marine sponges were immediately used for phylogenetic analyses of electrically retrieved microorganisms. The remaining marine sponges in the tubes were stored at −80 °C until use.

### Electrode Preparation

**Fig. 1:**
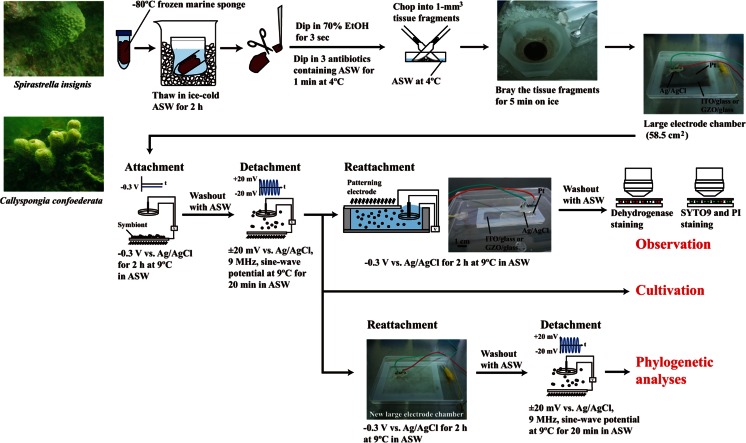
Schematic illustration of the electrical retrieval method for sponge-associated microorganisms in cryopreserved marine sponges. Photographs of the black homogenized tissue are from *S. insignis. ASW* artificial seawater

A large electrode chamber device was constructed for collecting microorganisms and performing molecular phylogenetic analyses (Koyama et al. 2013). A 110 × 85-mm^2^ and 5-mm-thick silicon rubber plate with a hollow interior measuring 90 × 65 mm^2^ was glued to either a 125 × 85-mm^2^ plane indium tin oxide/glass (ITO) or a gallium-doped zinc oxide/glass (GZO) electrode with silicon bonding. The ITO or GZO electrode was placed at the bottom of the chamber device and housed in a sterile square plastic dish (Fig. 1, upper right photograph). A 30-mmϕ Pt ring counterelectrode and a Ag/AgCl reference electrode were placed on the plastic lid of the square plastic dish.

A patterned electrode chamber device was used for a second analysis of the resistance of electrically attached microorganisms against gravitational force (Koyama et al. 2013). Either a patterned working ITO electrode or patterned working GZO electrode was constructed by vacuum evaporation of either indium tin oxide (<10 Ω/cm^2^) or gallium-doped zinc oxide (<10 Ω/cm^2^) and an insulator of silicon dioxide (SiO_2_) onto 76 × 26-mm^2^ silica glass plates (1 mm thick) (Geomatec Co., Ltd., Yokohama, Japan). Detailed patterning of the reticulated optically transparent working electrode with arrayed square glass regions was described elsewhere (Koyama 2011; Koyama et al. 2013). The 76 × 26-mm^2^ and 5-mm-thick silicon rubber plate with a hollow interior measuring 66 × 16 mm^2^ was glued to the 76 × 26-mm^2^ slide glass by silicon bonding. The patterned ITO or patterned GZO electrode was attached to the top of the silicon rubber box (Fig. 1, middle photograph). The fabricated silicon rubber box was housed in a sterile square plastic dish. A 12-mmϕ Pt ring counterelectrode and a Ag/AgCl reference electrode were placed on the plastic lid of the square plastic dish.

Both the patterned and large working electrodes were sonicated in ultrapure water for 5 min, immersed in 1 M NaOH for 5 min to remove any unwanted deposits, and then washed with ultrapure water and dried. Then, the three electrode chambers were irradiated with UV light for 5 min for sterilization.

### Potential Application

Constant and 9-MHz sine-wave potentials were applied to the optically transparent working electrode using the Ag/AgCl reference and the Pt counterelectrode (Koyama 2011; Koyama et al. 2013). The constant potential application was monitored with a potentiostat (PS-14, Toho Technical Research, Tokyo, Japan). The ±20-mV vs. Ag/AgCl, 9-MHz sine-wave potential application for cell detachment was performed with an electrochemical cell detachment system (Able Co., Ltd., Tokyo, Japan). The output potentials were confirmed using a digital oscilloscope (MT-770, Xiamen Lilliput Technology Co., Ltd., Fujian, China).

### Electrical Retrieval of Sponge-Associated Microorganisms

The ER method for sponge-associated microorganisms from cryopreserved marine sponges is schematically illustrated in Fig. 1. To obtain the sponge-associated microorganisms, we carefully removed any microbial contaminants attached to the surface of the sponge tissue using previously described deep-sea animal cell and tissue culture techniques (Koyama and Aizawa 2000, 2008; Koyama et al. 2003, 2005; Koyama 2007). The frozen marine sponge tissue was transferred into ice-cold sterile artificial seawater (30 g of NaCl, 0.7 g of KCl, 5.3 g of MgSO_4_⋅7H_2_O, 10.8 g of MgCl_2_⋅6H_2_O, 1 g of CaCl_2_⋅2H_2_O L^−1^) and incubated for 2 h. The thawed or fresh marine sponge tissue was rinsed with fresh sterile artificial seawater cooled to 4 °C. The rinsed tissue was dipped into 70 % ethanol for 3 s and transferred into sterile artificial seawater (4 °C) containing 1 % (*v*/*v*) antibiotics (antibiotic antimycotic solution [100×], Sigma, St. Louis, MO, USA) for 1 min to prevent microbial contamination. After sterilization, the tissue was placed in a biological safety cabinet and rinsed again with fresh sterile artificial seawater cooled to 4 °C. No particles were detected inside the safety cabinet by an airborne particle counter (HHPC3+, Beckman Coulter Inc., Brea, CA, USA). In addition, the ER method was performed in a clean room with 0.5–1.0-μmϕ particles of less than 200 counts/L (HHPC3+, Beckman Coulter Inc.). The sterilized tissue was minced into 1-mm^3^ pieces in fresh sterile artificial seawater at 4 °C. The 1-mm^3^ tissue fragments were centrifuged for 2 min at 2150×*g* at 4 °C, and the excess artificial seawater was discarded. The tissue pieces were transferred to a sterilized quartz mortar and brayed for 5 min on ice (Fig. 1, upper-middle photograph). In negative-control experiments, the homogenate samples suspended in fresh sterile artificial seawater were pretreated with autoclaving for 15 min at 121 °C. After autoclaving, the homogenate was centrifuged for 2 min at 2150×*g* at 4 °C and the excess artificial seawater was discarded. After these procedures, the tissue homogenate samples were resuspended in fresh sterile artificial seawater at 4 °C. Then, 12.5 mL of each suspended homogenate was poured into separate large chamber devices. A −0.3-V vs. Ag/AgCl constant potential was applied to the large electrode for 2 h at 9 °C. We observed a weak electrical current of −0.1 to −0.3 μA/cm^2^ when a −0.3-V vs. Ag/AgCl negative constant potential was applied to the homogenate samples. After a 2-h application, the large electrode was washed three times with sterile artificial seawater at 4 °C. After washing the electrode surface, the microorganisms attached to the large electrode were detached by applying a ±20-mV vs. Ag/AgCl, 9-MHz sine-wave potential for 20 min in 12.5 mL of fresh sterile artificial seawater at 4 °C. For cultivation tests (Fig. 1), the detached microorganisms were collected with a cell scraper and cultured after the sine-wave potential application. The microorganisms were transferred to either a new large electrode chamber device or patterned electrode chamber device, and a −0.3-V vs. Ag/AgCl constant potential was applied for a further 2 h at 9 °C in sterile artificial seawater. After that application, each electrode was washed three times with sterile artificial seawater at 4 °C and observed under an epifluorescence microscope (BX51, Olympus, Tokyo, Japan) (Fig. 1). For molecular phylogenetic analyses (Fig. 1), the microorganisms on the large electrode were detached by application of a ±20-mV vs. Ag/AgCl, 9-MHz sine-wave potential for a further 20 min in 12.5 mL of fresh sterile artificial seawater at 4 °C. The electrically collected microorganisms were used in the phylogenic analyses.

### Fluorescence Microscopic Observation

To analyze the respiratory activity of the microorganisms attached to the electrode, we used a Bacstain CTC rapid staining kit for microscopy (Dojindo, Kumamoto, Japan). Cyano-ditolyl-tetrazolium chloride (CTC), a monotetrazolium redox dye that produces red fluorescent formazan when it is chemically or biologically reduced as in the presence of dehydrogenase activity, was used an indicator of respiration (Hiraishi and Yoshida 2004; Frederiks et al. 2006). The fluorescent formazan was exclusively localized on the surface of individual cells and not at intracellular sites (Frederiks et al. 2006). The respiratory activity staining solution was comprised of 20 μL of 50 mM CTC solution and 5 μL of enhancing reagent B added to 1 mL of sterile artificial seawater. After vortexing the staining solution, the microorganisms on the electrode were incubated with the staining solution at 9 °C for 30 min and observed using the epifluorescence microscope system with UPlan FL ×10/0.30 Ph1 and UPlan Fl N ×100/1.30 oil Ph3 objective lenses.

We distinguished living and dead sponge-associated microorganisms using a live/dead backlight bacterial viability kit for microscopy and quantitative assays (L7012, Molecular Probes, Eugene, OR, USA) under the epifluorescence microscope system according to the manufacturer’s recommendations. To obtain dead sponge-associated microorganisms to act as a benchmark, we prepared sponge homogenates with either 70 % EtOH for 60 min at 60 °C or 0.1 % (*v*/*v*) Tween 20 (Wako, Osaka, Japan) containing artificial seawater for 5 min at 25 °C. To determine the survival rate in the homogenized tissues, 50 μg of the marine sponge homogenate was diluted with and then incubated in 1 mL of sterile artificial seawater containing 1.5 μL of SYTO9 and 1.5 μL of propidium iodide (PI) at 9 °C for 30 min. The green fluorescent nucleic acid staining probe SYTO9 detects microorganisms with intact cell membranes, whereas PI, a red fluorescent nucleic acid staining probe, only penetrates microorganisms with damaged cell membranes, causing a reduction in the SYTO9 green fluorescence and emitting red fluorescence. After these procedures, we counted the living and dead cells of sponge-associated microorganisms in the diluted homogenates using a hemocytometer and the epifluorescence microscope system (BX51, Olympus). To analyze survival rates of the sponge-associated microorganisms on the electrodes, the stained microorganisms were examined in random areas and more than 100 cells were counted in each test. No autofluorescence of the marine sponge-associated microorganisms was observed under blue excitation (WIB excitation 460–490 nm) light. The homogenized marine sponge tissue fractions have a small amount of vanishing green autofluorescence under WIB excitation light.

### Culture of Sponge-Associated Microorganisms

An originally formulated medium consisting of a mixture of the autoclaved microbial media and filter-sterilized nerve cell culture medium was used to cultivate sponge-associated microorganisms under aerobic conditions. The medium was composed of 1.8 % (*w*/*v*) Daigo’s artificial seawater SP for marine microalgae medium (Nihon Pharmaceutical Co., Ltd., Tokyo, Japan), 2 % (*w*/*v*) Bacto agar (Becton Dickinson and Company [BD], Sparks, MD, USA), 0.01 % (*w*/*v*) dextrose (Wako), 0.01 % (*w*/*v*) sucrose (Wako), 0.01 % (*w*/*v*) levulose (Wako), 0.01 % (*w*/*v*) malt sugar (Wako), 0.01 % (*w*/*v*) d(+)-galactose (Wako), 0.01 % (*w*/*v*) sorbit (Wako), 0.01 % (*w*/*v*) yeast extract (BD), 0.01 % (*w*/*v*) malt extract (BD), 0.01 % (*w*/*v*) tryptone (BD), 0.01 % (*w*/*v*) tryptic soy broth without dextrose (BD), 0.01 % (*w*/*v*) neopeptone enzymatic digest of protein (BD), 0.01 % (*w*/*v*) collagen peptide (Wako), 0.01 % (*w*/*v*) DMEM/F12 medium (Gibco by Life Technologies, Carlsbad, CA, USA), 0.01 % (*v*/*v*) fetal bovine serum (MP Biomedicals, Solon, Ohio, USA), and 0.01 % (*w*/*v*) horse serum (Gibco). Filter-sterilized nerve cell culture medium containing the collagen peptide, DMEM/F12 medium, and animal sera were added to the solution of the remaining autoclaved ingredients when the temperature had cooled to 65 °C or lower.

### Amplification of Microbial Small Subunit rRNA Gene and Pyrosequencing

Next-generation sequence analyses were conducted using the Ion Torrent Personal Genome Machine (PGM; Life Technologies Corp., Carlsbad, CA, USA) (Yergeau et al. 2012; Bondici et al. 2013). DNA was extracted from both the homogenized sponge liquid and the electrically retrieved microbes with the ER method using a PurElute Bacterial Genomic Kit (Edge Biosystems, Gaithersburg, MD, USA). PCR amplification of microbial small subunit (SSU) ribosomal RNA (rRNA) gene fragments using the primer set of “530F” and “907R” described previously (Nunoura et al. 2012) was conducted under the following conditions. The PCR mixture contained 0.5 volumes of 2 × GC buffer I for LA *Taq* polymerase (TaKaRa Bio Inc., Otsu, Japan), 0.1 U μL^−1^ of LA *Taq*, 0.4 pmol μL^−1^ of each primer mixture, and 1.6 nmol μL^−1^ of dNTP. The DNA amplification conditions were as follows: 2 min of denaturation at 96 °C; 25 cycles at 96 °C for 25 s, 50 °C for 30 s, and 72 °C for 45 s; and a final elongation step of 7 min at 72 °C. Amplified SSU rRNA gene fragments purified by agarose gel electrophoresis were subjected to end-repair treatment, followed by the linkage of the barcode adaptors with Ion Xpress Barcode Adaptors Kits (Life Technologies). The barcode-tagged amplicons were purified using the MonoFas DNA purification kit according to the manufacturer’s instructions and quantified using a Bioanalyzer and a quantitative fluorescent PCR method with a 7500 Real Time PCR System (PE Applied Biosystems, Foster City, CA, USA). Pooled, barcode-tagged SSU amplicons were sequenced using the Ion Torrent PGM and a 318 chip. The sequences retrieved from Ion Torrent analyses were processed using CLC Genomics Workbench ver. 7.01 (CLC Bio Japan Inc., Tokyo, Japan).

### Phylogenetic Assignment

Low-quality sequences with lower scores and shorter than 300 bp were removed using the Pipeline Initial Process tool on CLC Genomics Workbench (ver.6.01, CLC Bio Japan). Sequences passing through the quality check were aligned using the partial order algorithm (POA) (SINA, http://www.arb-silva.de/aligner/) with a reference multiple alignment SILVA SSU Ref NR (ver.108, http://www.arb-silva.de/). Then, tags were clustered into operational taxonomic units (OTUs) with 98.5 % sequence identity using MOTHUR v.1.31.0 with default parameters (Schloss et al. 2009), and subsequently, the taxonomic position of each OTU was automatically assigned based on BLAST analysis using SILVA Ref NR as a reference dataset of SSU rRNA gene sequences. Sequences with a relatively high E-value (>1.0E-30) or low identity (<90 %) to match the reference sequence best were designated as other archaea or bacteria, and sequences that did not show significant identity with any reference sequence were excluded from the analysis.

### Statistical Analysis

Statistical analysis was performed using Student’s *t* test. Calculations were performed using Microsoft Excel.

### Accession Numbers

Sequences and quality from ion torrent sequencer runs were deposited in the DDBJ/EMBL/GenBank database under accession numbers DRA002827 to DRA002830.

## Results

### Electrical Retrieval of Living Microorganisms from Frozen *S. insignis*

The total cell number, intact cell number, and survival rate of the microorganisms in thawed *S. insignis* samples after freezing at −80 °C were 4.2 ± 0.2 × 10^8^ cells/g tissue, 3.1 ± 0.4 × 10^8^ cells/g tissue, and 75 ± 7 % (mean ± SEM, *n* = 4), respectively (Table 1). After confirming that numerous living microorganisms could be detected in frozen *S. insignis* sponge tissue using the SYTO9 and PI double-staining test, we examined the electrical retrieval of sponge-associated microorganisms from frozen *S. insignis* tissue in subsequent experiments (Table 1). In all experiments, the constant potential applied to the optically transparent electrode was set at −0.3-V vs. Ag/AgCl because we demonstrated that a phylogenetically wide range of living microorganisms dispersed in artificial seawater were attached to the potential-applied electrode (Koyama et al. 2013). A potential of −0.4-V vs. Ag/AgCl or less induced microbial damage due to the adsorption wave of positive ions in the artificial seawater (Koyama et al. 2013). After duplicate retrievals, we obtained 1.7 ± 0.4 × 10^7^ cells of sponge-associated microorganisms/58.5 cm^2^ from the ITO electrode (mean ± SEM; *n* = 12 of 3 independent experiments) from the homogenized *S. insignis* tissue in the present study (Table 1).

**Table 1:** Sponge-associated microorganisms from homogenized tissues

	*S. insignis*	*C. confoederata*
Membrane-intact cells^a^	3.1 ± 0.4 (×10^8^ cells/g tissue)	2.0 ± 0.1 (×10^8^ cell/g tissue)
Total cells^a^	4.2 ± 0.2 (×10^8^ cells/g tissue)	8.8 ± 0.9 (×10^8^ cell/g tissue)
Estimated survival rate^a^	75 ± 7 (%)	23 ± 2 (%)
Electrical retrieved intact cells^b^	1.7 ± 0.4 (×10^7^ cells/58.5 cm^2^ electrode)	4.6 ± 0.4 (×10^6^ cells/58.5 cm^2^ electrode)

The sponge-associated microorganisms were retrieved from thawed homogenized stored at −80 °C. The microorganisms were stained with both SYTO9 and PI

^a^We counted the sponge-associated microorganisms in the diluted homogenate using a hemocytometer and a fluorescence microscope. The values are mean ± SEM (*n* = 4)

^b^Each 1.5–7.5-g sample of homogenized *S. insignis* sponge tissue was placed in a large ITO electrode chamber; 2.0-g sample of homogenized *C. confoederata* tissue was placed in a large ITO electrode chamber. The electrical retrieval of the microorganisms was sequentially performed twice with each unused large ITO electrode chamber device (Fig. 1). The retrieved microorganisms were counted using a hemocytometer. In *S. insignis,* the values are mean ± SEM (*n* = 12 of 3 independent experiments). In *C. confoederata,* the values are mean ± SEM (*n* = 4)

**Fig. 2:**
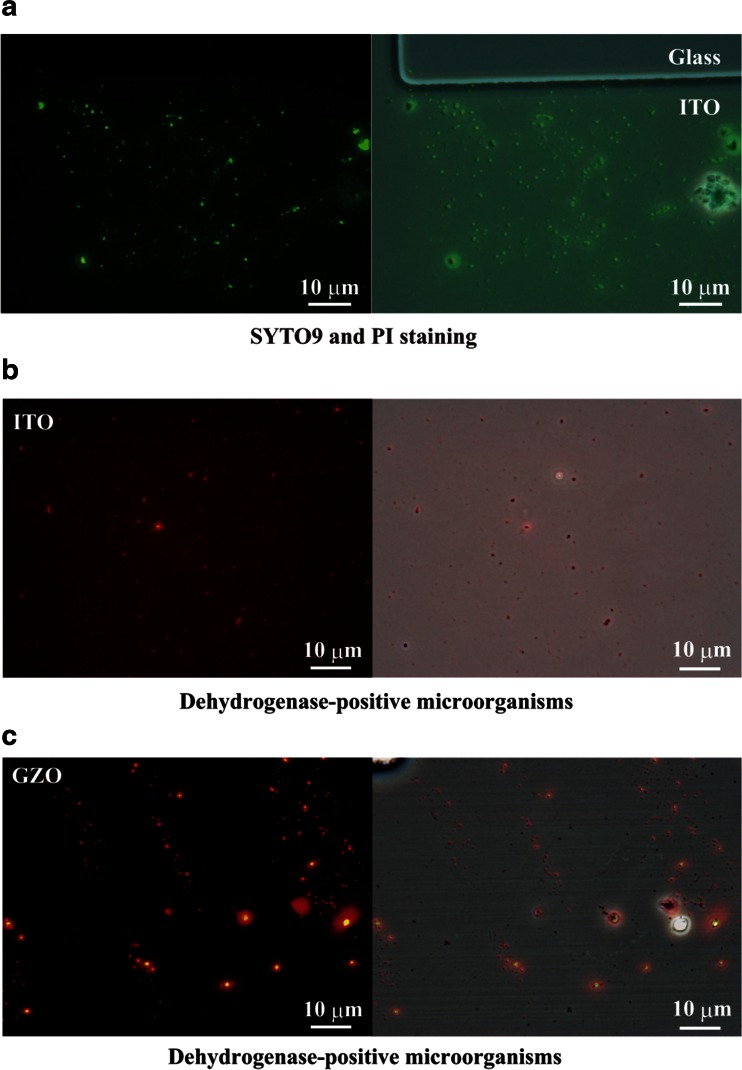
*S. insignis*-associated microorganisms on the −0.3-V vs. Ag/AgCl applied optically transparent electrodes. To avoid nonspecific adsorptions, two electrical retrievals were conducted using the first large and the second top patterned electrodes, respectively. **a** The microorganisms stained with both SYTO9 and PI on the top patterned ITO electrode surface. The microorganisms were examined in random areas and assayed by counting the percentage of living cells. The survival rate of the microorganisms on the electrode was 100 % (531 of 531 cells). **b** Dehydrogenase-positive microorganisms on the patterned ITO electrode surface. **c** Dehydrogenase-positive microorganisms on the patterned GZO electrode surface

To investigate whether the electrically retrieved sponge-associated microorganisms had a higher survival rate than those from homogenized frozen tissue, two sequential electrical retrievals using the first large and the second top patterned working electrodes were performed in *S. insignis* (Figs. 1 and 2). All (531 of 531 cells) of the sponge-associated microorganisms from *S. insignis* had intact cell membranes on the −0.3-V potential-applied ITO electrode in the double-staining test (Fig. 2a). We found that only the living cells were collected and the dead cells were washed away by the ER method (Table 1 and Fig. 2a). The survival rate of the sponge-associated microorganisms from *S. insignis* was almost 100 % after the ER method (Fig. 2a).

We also determined whether the membrane-intact sponge-associated microorganisms had respiratory activity. The attached microorganisms from the homogenized *S. insignis* tissue on the optically transparent electrodes were stained with CTC as an indicator of respiration (Frederiks et al. 2006; Hiraishi and Yoshida 2004). Sponge-associated dehydrogenase-positive microorganisms attached to both the −0.3-V vs. Ag/AgCl potential-applied ITO and GZO electrodes (Fig. 2b, c). The results in Fig. 2 indicate that the −0.3-V potential-applied electrode attracted living sponge-associated microorganisms against gravitational force.

**Fig. 3:**
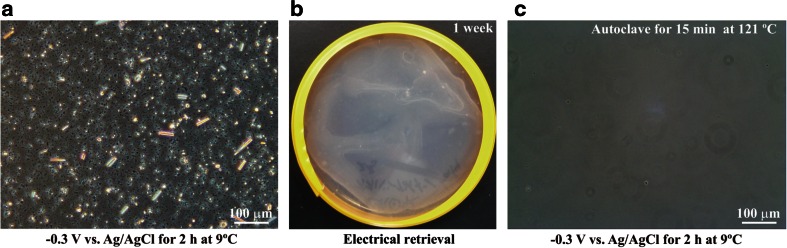
Cultivation of *S. insignis*-associated microorganisms detached from the ITO electrode. To attach the sponge-associated microorganisms to the ITO electrode, the −0.3-V vs. Ag/AgCl potential was applied for 2 h at 9 °C. After a 2-h application, the electrode was washed three times with sterile artificial seawater at 4 °C and observed using a phase-contrast microscope. After washing, the microorganisms on the electrode were detached by ±20-mV vs. Ag/AgCl, 9-MHz sine-wave potential application for 20 min at 9 °C. **a** Electrical attachment of *S. insignis*-associated microorganisms. The homogenized sponge tissue was seeded at a density of 27 mg/cm^2^ and the −0.3-V vs. Ag/AgCl potential was applied for 2 h at 9 °C to the ITO electrode. **b** Cultivation of *S. insignis*-associated microorganisms for 1 week at room temperature. The microorganisms on the electrode were detached by ±20-mV vs. Ag/AgCl, 9-MHz sine-wave potential application for 20 min at 9 °C. The detached microorganisms were seeded on agar plates. **c** Electrical attachment of *S. insignis*-associated microorganisms pretreated with autoclaving at 121 °C for 15 min. After autoclaving, the homogenized tissue was seeded at a density of 50 mg/cm^2^ and applied to the −0.3-V vs. Ag/AgCl potential for 2 h. Few or no sponge-associated microorganisms were observed on the ITO electrode surface

Next, we investigated whether the electrically retrieved microorganisms included culturable microorganisms using an originally formulated medium composed of microbial media and animal nerve cell culture medium. Figure 3a shows the −0.3-V vs. Ag/AgCl potential-induced electrical attachment of microorganisms from homogenized *S. insignis* tissue on the ITO electrode surface. We confirmed that the electrically attached microorganisms could be cultured on agar medium after detachment from the ITO electrode by high-frequency wave potential application (Figs. 1 and 3b). When the *S. insignis* tissue homogenates were pretreated with autoclaving for 15 min at 121 °C, few or no microorganisms were attached to the ITO electrode (Fig. 3c). The results in Figs. 2 and 3 and Table 1 clearly show that the −0.3-V vs. Ag/AgCl potential induced the attachment of living microorganisms derived from *S. insignis* tissue frozen at −80 °C.

### Analyses of Electrically Retrieved Microbiota from *S. insignis* and *C. confoederata*

**Fig. 4:**
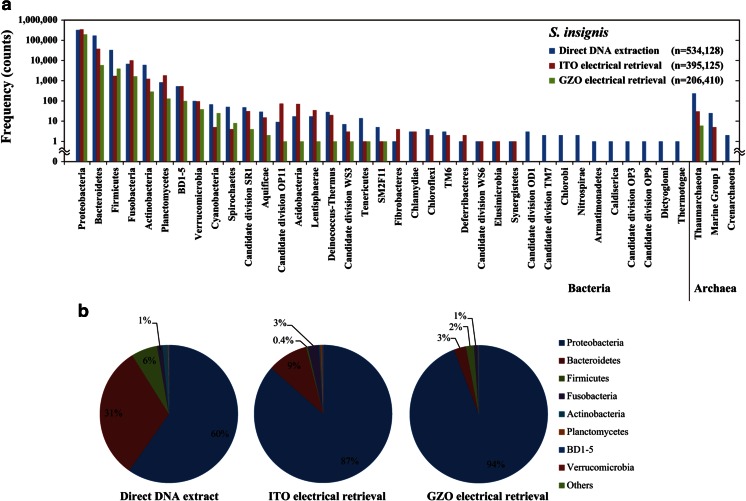
Phylogenetic affiliation of strains isolated from the marine sponge *S. insignis*. In the GZO electrode retrievals, a fresh specimen was immediately used for phylogenetic analysis. Thawed samples and those that had been frozen at −80 °C were used in the ITO electrode retrievals and direct DNA extractions, respectively. **a** *Bar chart* and **b** *circle chart* of microbial frequency distributions

In the next series of experiments, we determined which types of microorganisms would attach to the ITO and GZO electrode surfaces with the −0.3-V vs. Ag/AgCl constant potential application (Figs. 1 and 4, Table 2). The electrical retrieval of the microorganisms was sequentially performed twice in each experiment (Fig. 1). The electrically retrieved microorganisms with the ITO and the GZO electrodes were compared with those in the original crude samples by phylotype analysis of PCR-amplified 16S rRNA genes (Fig. 4 and Table 2). We used homogenized frozen *S. insignis* tissue for the phylogenetic analyses in both the ITO electrode retrievals and direct DNA extraction (Fig. 4 and Table 2). In the GZO electrode retrievals, a fresh specimen of *S. insignis* was used for the phylogenetic analyses (Fig. 4 and Table 2). Figure 4 shows the microbiota of *S. insignis* after ITO and GZO electrical retrievals and direct DNA extraction. More than 99.9 % of the electrically collected microorganisms had the same composition at the phylum and class levels between frozen *S. insignis* with the ITO electrode and fresh *S. insignis* with the GZO electrode retrievals (Fig. 4 and Table 2). In addition, 99.9 % or more of the phylotypes among the microorganisms electrically retrieved using the two methods were common to the gene clones from direct DNA extraction (Fig. 4 and Table 2). Microorganisms associated with *S. insignis* belonging to 29 phyla and 70 classes were obtained using the ITO electrical retrieval method (Fig. 4 and Table 2). Using the GZO electrodes, we obtained 20 phyla and 35 classes of sponge-associated microorganisms (Fig. 4 and Table 2). The results indicated little phylogenetic difference between the microbial composition retrieved electrically and with direct DNA extraction.

**Table 2:** Analysis of 16S rRNA sequencing reads from marine sponges *C. confoederata* and *S. insigis*

Kingdom	Phylum	Class	*C. confoedarate* ^a^		*S. insignis* ^b^		
			Direct extraction	ITO electrical retrieval	Direct extraction	ITO electrical retrieval	GZO electrical retrieval
Bacteria	*Acidobacteria*	Acidobacteria	0	0	8	6	1
		Holophagae	1	5	39	25	0
		RB25	0	0	1	0	0
	*Actinobacteria*	Acidimicrobiia	2	21	240	142	0
		Actinobacteria	11	93	280	390	288
		Thermoleophilia	0	0	11	1	0
	*Aquificae*	Aquificae	0	1	3	2	2
	*Armatimonadetes*	Unclassified	0	0	1	0	0
	*Bacteroidetes*	Bacteroidia	5	46	98	52	687
		Cytophagia	7	33	203	396	12
		Flavobacteria	6996	42,283	166,586	36,377	5128
		Sphingobacteria	6	119	323	508	40
		AMV16	0	0	0	1	0
		BD2-2	1	2	17	4	1
		DUNssu192	0	2	34	7	0
		Ika33	0	0	1	0	0
		SB-1	0	0	1	0	0
		VC2.1 Bac22	0	2	1	1	0
		Class *incertae sedis*	6	19	56	51	0
	BD1-5	Unclassified	0	4	17	35	98
	*Caldiserica*	Caldisericia	0	0	1	0	0
	*Candidate division* OD1	Unclassified	0	1	1	4	0
	*Candidate division* OP3	Unclassified	0	1	2	0	0
	*Candidate division* OP9	Unclassified	0	0	1	0	0
	*Candidate division* OP11	Unclassified	1	0	2	0	1
	*Candidate division* SR1	Unclassified	0	0	1	2	4
	*Candidate division* TM7	Unclassified	0	1	14	1	0
	*Candidate division* WS3	Unclassified	0	0	0	0	1
	*Candidate division* WS6	Unclassified	1	0	3	0	0
	*Chamydiae*	Chalamydiae	2	8	67	5	0
	*Chlorobi*	Chlorobia	0	0	4	2	0
	*Chloroflexi*	Anaerolineae	0	1	2	9	0
		Caldilineae	0	1	8	58	0
		Chloroflexi	0	0	3	0	0
		Ktedonobacteria	0	0	0	3	0
		Thermomicrobia	0	0	1	0	0
		GIF9	0	0	1	0	0
		JG37-AG-4	0	0	1	0	0
		SAR202 clade	0	0	1	0	0
	*Cyanobacteria*	Chloroplast	32	81	790	1742	23
		Acaryochloris	0	0	1	1	0
		Subsection I	0	0	7	5	1
		Subsection II	0	1	4	12	1
		Subsection III	0	2	13	28	0
		4C0d-2	0	0	2	0	0
		ML635J-21	0	0	2	0	0
		SHA-109	0	0	2	0	0
		SM2F09	0	0	0	1	0
		WD272	0	0	0	1	0
	*Deferribacteres*	Deferribacteres	0	8	1	1	0
	*Deinococcus-Thermus*	Deinococci	0	11	9	73	1
	*Dictyoglomi*	Dictyoglomia	0	0	1	0	0
	*Elusimicrobia*	Elusimicrobia	0	0	1	1	0
	*Fibrobacteres*	Fibrobacteria	0	0	5	1	0
	*Firmicutes*	Bacilli	26	251	6528	10,064	3481
		Clostridia	6	30	156	38	470
		Erysipelotrichi	0	0	2	0	2
	*Fusobacteria*	Fusobacteria	9	81	98	94	1638
	*Lentisphaerae*	Lentisphaeria	1	16	28	20	1
	*Nitrospirae*	Nitrospira	0	0	3	3	0
	*Planctomycetes*	Planctomycetacia	1073	2386	32,832	1585	124
		Phycisphaerae	2	69	113	60	3
		Pla3 lineage	0	0	0	4	0
		Pla4 lineage	0	0	1	0	0
		OM190	1	4	11	63	2
		028H05-P-BN-P5	0	0	1	4	0
		BD7-11	0	0	2	4	0
		MD2896-B258	0	0	1	0	0
		VadinHA49	0	0	0	1	0
	*Proteobacteria*	Alphaproteobacteria	2178	22,609	66,029	11,375	933
		Betaproteobacteria	3237	15,995	114,702	9542	79,052
		Deltaproteobacteria	14	80	260	134	4
		Epsilonproteobacteria	2058	4058	61,853	4080	678
		Gammaproteobacteria	2425	95,634	76,141	316,605	113,672
		ARKDMS-49	0	0	2	0	0
		ARKICE-90	0	80	53	29	5
		Elev-16S-509	0	5	6	5	0
		JTB23	1	9	35	16	0
		MACA-EFT26	0	1	1	3	0
		pltb-vmat-80	1	0	3	1	0
		SC3-20	4	23	61	129	4
		SK259	0	0	1	0	0
		SPOTSOCT00m83	1	0	3	14	0
		TA18	1	2	40	5	0
	RsaHF231	Unclassified	0	0	1	0	0
	SM2F11	Unclassified	0	0	0	0	1
	*Spirochaetes*	Spirochaetes	0	0	7	3	8
	*Synergistetes*	Synergistia	0	0	1	1	0
	TA06	Unclassified	0	1	0	0	0
	*Tenericutes*	Unclassified	0	14	29	15	1
	*Thermotogae*	Thermotogae	0	0	2	0	0
	TM6	Unclassified	0	2	50	4	0
	*Verrucomicrobia*	Verrucomicrobiae	153	733	5912	1224	38
		Opitutae	0	7	26	13	0
		Spartobacteria	0	0	2	0	0
		OPB35 soil group	0	0	0	4	0
	WCHB1-60	Unclassified	0	0	1	0	0
	Subtotal		18,262	184,836	533,868	395,090	206,404
Archaea	*Crenarchaeota*	Thermoprotei	0	1	2	0	0
	*Marine Group I*	Unclassified	0	12	25	5	0
	*Thaumarchaeota*	*incertae sedis*	6	81	233	30	6
	Subtotal		6	94	260	35	6
	Total		18,268	184,930	534,128	395,125	206,410

In the GZO electrode retrievals, a fresh specimen was immediately used for the phylogenetic analysis

^a^Twenty-four phyla and 51 classes of bacteria and 3 phyla and 3 classes of archaea were obtained from *C. confoederata*

^b^Thirty-nine phyla and 96 classes of bacteria and 3 phyla and 3 classes of archaea were obtained from *S. insignis*

**Fig. 5:**
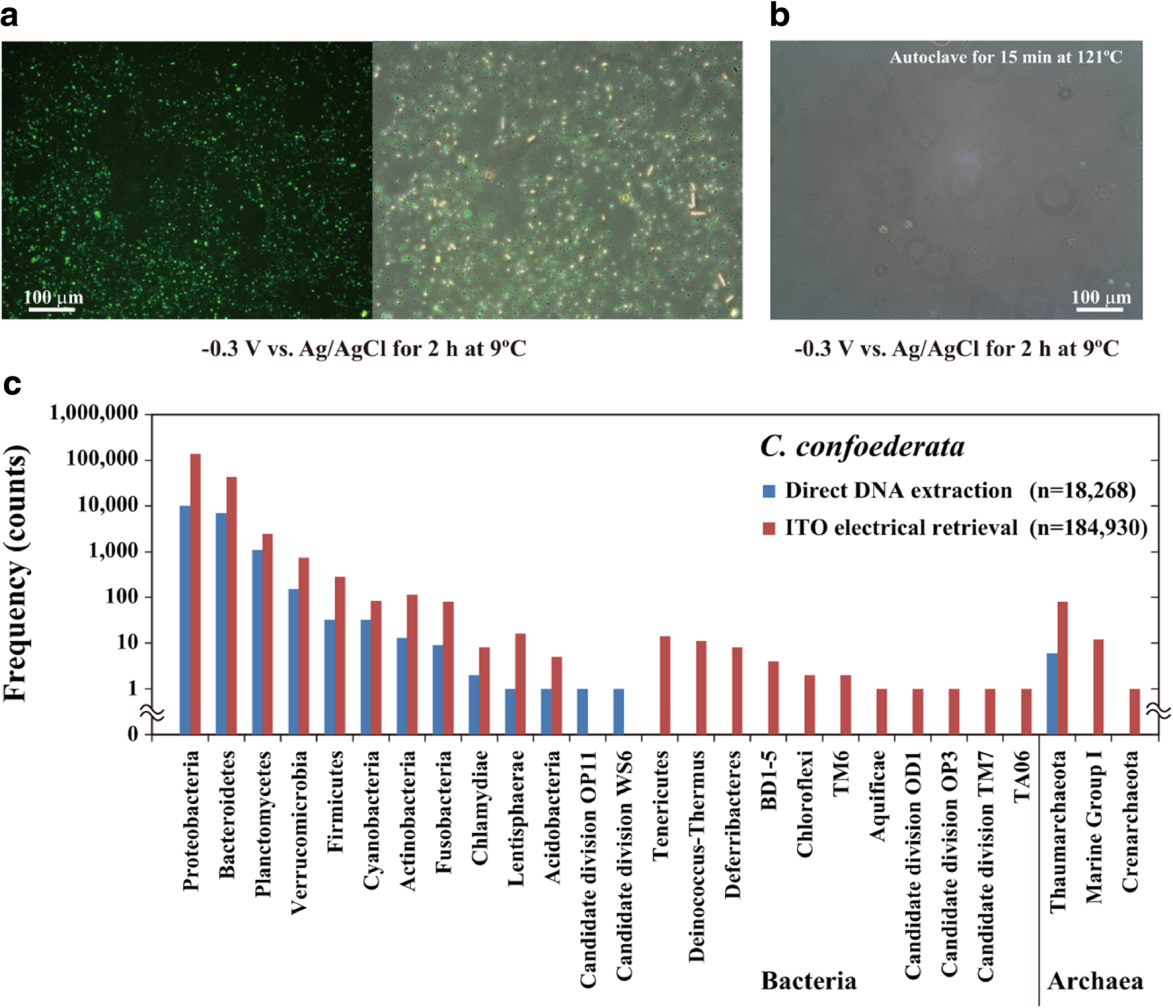
Electrical retrieval of *C. confoederata*-associated microorganisms. **a** The microorganisms from *C. confoederata* stained with both SYTO9 and PI on the first ITO electrode surface. The microorganisms were examined in random areas and assayed by counting the percentage of living cells. The survival rate of the microorganisms on the first ITO electrode was 99.6 % (500 of 502 cells). **b** First electrical attachment of *C. confoederata*-associated microorganisms pretreated with autoclaving at 121 °C for 15 min. After autoclaving, the homogenized tissue was applied to the −0.3-V vs. Ag/AgCl potential for 2 h. Few or no sponge-associated microorganisms were observed on the first ITO electrode surface. **c** Phylogenetic affiliation of strains isolated from the marine sponge *C. confoederata*. Thawed samples and those that had been frozen at −80 °C were used in the ITO electrode retrievals and direct DNA extractions, respectively

Next, *C. confoederata* frozen at −80 °C was also examined to compare the microbial compositions of living microorganisms retrieved electrically and with direct DNA extraction (Table 1, Figs. 1 and 5). The total cell number, intact cell number, and survival rate of the microorganisms in thawed *C. confoederata* samples after freezing at −80 °C were 2.0 ± 0.1 × 10^8^ cells/g tissue, 8.8 ± 0.9 × 10^8^ cells/g tissue, and 23 ± 2 % (mean ± SEM, *n* = 4), respectively (Table 1). We obtained 4.6 ± 0.4 × 10^6^ cells of sponge-associated microorganisms/58.5 cm^2^ from the ITO electrode (mean ± SEM; *n* = 4) from the homogenized *C. confoederata* tissue after the duplicate retrievals (Table 1). The microorganisms from homogenized frozen *C. confoederata* tissue attached to the −0.3-V potential-applied ITO electrode had intact cell membranes and a survival rate of 99.6 % (500 of 502 cells) in the SYTO9 and PI double-staining test (Fig. 5a). When the *C. confoederata* tissue homogenates were pretreated with autoclaving for 15 min at 121 °C, few or no microorganisms attached to the −0.3-V potential-applied electrode (Fig. 5b). The results in Fig. 5a, b and Table 1 indicate that only living cells were collected and dead cells were removed after the ER method. Figure 5c compares the electrical retrieval and direct DNA extraction of microbiota of *C. confoederata*. Microorganisms associated with *C. confoederata* belonging to 25 phyla and 50 classes were obtained using the electrical retrieval method (Figs. 1 and 5, Table 2). Nearly all (99.9 % or more) of the relative abundance at the phylum and class levels among the electrically retrieved microorganisms was common to the gene clones from direct DNA extraction (Fig. 5 and Table 2).

We electrically retrieved 32 phyla and 72 classes of bacteria and 3 archaea of *Crenarchaeota thermoprotei*, *Marine Group I*, and *Thaumarchaeota incertae sedis* from *S. insignis* and *C. confoederata* (Figs. 4 and 5, Table 2). The majority of the electrically retrieved clones in both sponges were affiliated with Proteobacteria. Proteobacteria comprised 87, 94, and 75 % of the total microorganisms in *S. insignis* with ITO and GZO electrode and *C. confoederata* with ITO electrode retrieval, respectively. These Proteobacteria were mainly composed of *alpha-*, *beta-*, *delta-*, *epsilon-*, and *gammaproteobacteria* in both sponges (Table 2). The remaining phyla in *S. insignis* were wide ranging and affiliated with *Acidobacteria*, *Actinobacteria*, *Aquificae*, *Bacteroidetes*, BD1-5, *Candidate division* OD1, OP11, SR1, TM7, WS3, *Chlamydiae*, *Chlorobi*, *Chloroflexi*, *Cyanobacteria*, *Deferribacteres*, *Deinococcus-Thermus*, *Elusimicrobia*, *Fibrobacteres*, *Firmicutes*, *Fusobacteria*, *Lentisphaerae*, *Nitrospirae*, *Planctomycetes*, SM2F11, *Spirochaetes*, *Synergistetes*, *Tenericutes*, TM6, and *Verrucomicrobia* in Bacteria, and *Marine Group I*, and *Thaumarchaeota* in Archaea (Fig. 4 and Table 2). In *C. confoederata*, the remaining phyla were affiliated with *Acidobacteria*, *Actinobacteria*, *Aquificae*, *Bacteroidetes*, BD1-5, *Candidate division* OD1, OP3, TM7, *Chlamydiae*, *Chloroflexi*, *Cyanobacteria*, *Deferribacteres*, *Deinococcus-Thermus*, *Firmicutes*, *Fusobacteria*, *Lentisphaerae*, *Planctomycetes*, TA06, *Tenericutes*, TM6, and *Verrucomicrobia* in Bacteria, and *Crenarchaeota*, *Marine Group I*, and *Thaumarchaeota* in Archaea. Sequences representing the bacterial phyla *Armatimonadetes*, *Caldiserica*, *Candidate division* OP9, *Dictyoglomi*, RsaHF231, *Thermotogae*, and WCHB1-60 were only present in the direct DNA extractions at relative abundance of 1 × 10^−3^ % (8 of 552,396 sequences, Table 2). Similarly, the bacterial phyla *Candidate division* WS3 and SM2F11 were only found in the electrical retrievals at relative abundance of 3 × 10^−4^ % (2 of 786,465 sequences, Table 2). The results demonstrated that the ER method can be used to collect a phylogenetically broad range of microorganisms which reflects the organization of the microbial community in marine sponges.

## Discussion

We electrically retrieved the living microorganisms from the frozen marine sponges *S. insignis* and *C. confoederata* using an optically transparent working electrode by respective applications of a −0.3-V vs. Ag/AgCl and a ±20-mV vs. Ag/AgCl, 9-MHz sine-wave potential in artificial seawater at 9 °C (Table 1, Figs. 2, 3, and 5). After the ER method, both the survival rates of the sponge-associated microorganisms were 99.6 % (500 of 502 cells) from *C. confoederata* and 100 % (531 of 531 cells) from *S. insignis*, respectively. Thirty-two phyla and 72 classes of bacteria and 3 archaea of *Crenarchaeota thermoprotei*, *Marine Group I*, and *Thaumarchaeota incertae sedis* were obtained when we applied the ER method to separate them from the marine sponges. More than 99.9 % of the relative abundance among electrically retrieved microorganisms was common to the gene clones from the direct DNA extractions. The ER method yielded a phylogenetically broad range of the living microorganisms, accurately reflecting the microbial community structure in the marine sponges (Figs. 4 and 5, Table 2). A small number of microbial phyla at the relative abundance of less than 0.1 % did not collect with both the ITO and GZO electrical retrievals (Table 2). Notably, the class of *Acidimicrobiia* in *S. insignis* was not collected by the GZO electrical retrieval in spite of 240 and 142 sequences were found by the direct DNA extraction and the ITO electrical retrieval (Table 2). The *Acidimicrobiia* might distinguish the ITO electrode surface due to specific adhesion proteins.

The ER method might be applicable not only to sponge-associated microorganisms but also to chemosymbiotic biological samples because the majority of the episymbiotic and endosymbiotic chemosynthetic bacterial clusters are within the *delta-*, *epsilon-*, and *gammaproteobacteria* (Stewart et al. 2005; Cavanaugh et al. 2006; Dubilier et al. 2008). Associations between sponges and microorganisms can be maintained over different generations in either of two ways: (1) microorganisms can be recruited from the surrounding water by filter feeding or (2) microbial symbionts can be transmitted between successive host generations via their fertilized eggs (Usher and Ereskovsky 2005; Ereskovsky et al. 2005). The ER method might also be utilized for studying the symbiont transmission mechanisms in marine sponges.

The ER method is a powerful tool to eliminate unnecessary microbiota and obtain rare environmental microorganisms by potential application of optimal wave shape, optimal resonance frequency, optimal amplitude, and optimal time length. We reduced unwanted microbiota and isolated the novel gram-negative strain *Brevundimonas denifitricans* from 1180-m deep-seafloor sediment 11 m below the seabed off the Shimokita Peninsula, Japan, using the ER method (Tsubouchi et al. 2014). We reported that microorganisms such as *E. coli* recognized and selectively attached to small regions of a −0.4-V vs. Ag/AgCl applied potential 5-μmϕ circular ITO microelectrode array at the single-cell level (Koyama et al. 2013). If the ITO microelectrode array uses a water-repellent insulating coat, the application of the ER method could be expanded to include single-cell cultivation technology, single microorganisms could be cultivated in a few microliters of medium when the cells attach to the 5-μmϕ circular ITO microelectrode with water-repellent insulation. Because electrically attached microorganisms are immobilized on a negative potential-applied electrode surface (Koyama et al. 2013), the medium of the single cells can be replaced, allowing the examination of a variety of chemical compositions. It is, therefore, practical to cultivate the sponge-associated microorganisms in media with varied compositions in order to screen for diverse biologically active compounds. Moreover, it is important to analyze and compare a small amount of the intracellular metabolites such as the precursors and the regulators before and after each of the microorganisms stops producing the bioactive compounds in the single-cell culture. We hoped that employment of the ER method will be possible in the future to develop the single-cell cultivation device of the sponge-associated microorganisms attached on the microelectrode array for genome, transcriptome, proteome, and metabolome analyses of the bioactive compounds producing symbionts.

Numerous living microbial cells were obtained from the large electrode in homogenized frozen marine sponge samples (Table 1, Figs. 2, 3, and 5). We found 4.2 ± 0.2 × 10^8^ cells/g tissue from *S. insignis* and 8.8 ± 0.9 × 10^8^ cells/g tissue from *C. confoederata* (Table 1), corresponding to 6.4 ± 4.6 × 10^8^ cells/g tissue from *Aplysinaaerophoba* and 1.5 × 10^8^–8.3 × 10^9^ cells/mL from *Rhopaloeides odorabile* (Friedrich et al. 2001; Webster and Hill 2001). These microbial numbers exceed those of seawater by about two to four orders of magnitude (Friedrich et al. 2001; Webster and Hill 2001). Marine sponges can be grouped according to the density of bacteria within their tissues into high-microbial abundance (HMA) and low-microbial abundance (LMA) sponges (Hentschel et al. 2003; Kennedy et al. 2014). HMA sponges were reported to have microbial densities of 10^8^–10^10^ bacteria g^−1^ of tissue, while LMA sponges have 10^5^–10^6^ bacteria g^−1^ of tissue (Hentschel et al. 2003; Kennedy et al. 2014). Based on the density of microorganisms within the tissue, the microbial communities of *S. insignis* and *C. confoederata* were typical of HMA sponges.

In our previous study, we showed that soil and sediment microorganisms can attach to the ITO electrode with a negative applied potential when the cells are suspended in nonnutritive media such as PBS(−), MOPS buffer, tricine buffer, and artificial seawater (Koyama et al. 2013). The results of this and our previous study (Koyama et al. 2013) suggest that the electrodes with a negative applied potential act as an energy source for a broad range of microorganisms. The −0.3-V vs. Ag/AgCl negative potential corresponds to −0.1-V of reduction potential (*E*_0_′). If the electrode acts as an electron donor in sterile artificial seawater, oxygen and heavy metal ions such as ferric iron and manganese ion would be electron acceptors. Further research will determine the mechanisms by which the negative potential induced attachment by a wide range of microorganisms.
